# Variable Metastatic Potentials Correlate with Differential Plectin and Vimentin Expression in Syngeneic Androgen Independent Prostate Cancer Cells

**DOI:** 10.1371/journal.pone.0065005

**Published:** 2013-05-22

**Authors:** Tanya C. Burch, Megan T. Watson, Julius O. Nyalwidhe

**Affiliations:** 1 Department of Microbiology and Molecular Cell Biology, Eastern Virginia Medical School, Norfolk, Virginia, United States of America; 2 Leroy T. Canoles Jr. Cancer Research Center, Eastern Virginia Medical School, Norfolk, Virginia, United States of America; University of Illinois, United States of America

## Abstract

Prostate cancer is a clinically heterogeneous disease, ranging from indolent asymptomatic disease to very aggressive metastatic and life threatening forms of the disease. Distant metastasis represents the major lethal cause of prostate cancer. The most critical clinical challenge in the management of the patients is identifying those individuals at risk of developing metastatic disease. To understand the molecular mechanisms of prostate cancer metastasis and identify markers with metastatic potential, we have analyzed protein expression in two syngeneic prostate cancer cells lines PC3-N2 and PC3-ML2 using isobaric tags for relative and absolute quantitation labeling and multi-dimensional protein identification technology liquid chromatography matrix assisted laser desorption ionization tandem mass spectrometry. PC3-N2 is lowly metastatic while PC3-ML2 highly metastatic. A total of 1,756 proteins were identified in the analyses with 130 proteins showing different expression levels (p<0.01) in the two cell lines. Out of these, 68 proteins were found to be significantly up-regulated while 62 are significantly down-regulated in PC3-ML2 cells compared with PC3-N2 cells. The upregulation of plectin and vimentin which were the most significantly differentially expressed were validated by Western blot and their functional relevance with respect to invasion and migration was determined by siRNA gene silencing. To our knowledge, this study is the first to demonstrate that up-regulation of vimentin and plectin expression positively correlates with the invasion and metastasis of androgen-independent PCA.

## Introduction

In Europe and the US, Prostate cancer (PCa) is the most prevalent cancer among men and the third most common cause of cancer related-deaths. In 2011 there were an estimated 240,890 new cases and 33,720 deaths from the disease in the US [1]. For the last 2 decades, early detection and screening of PCa has been mainly based on the detection of prostate specific antigen (PSA) in serum in addition to digital rectal examination (DRE), and histological assessment of transrectal ultrasound (TRUS) guided biopsy material [2]. Although most of the cases are detected at an early stage, the disease is clinically heterogeneous, ranging from indolent asymptomatic disease to very aggressive metastatic and life threatening forms of the disease. Over 7% of the cases detected eventually develop distant metastatic disease [3]. The prognosis for these men is poor and they have an average survival of 24 to 48 months [3]. The most critical clinical challenge for PCa disease management is to determine which of these two diverse forms of the disease a patient develops. The most common site for PCa metastasis is the bone; ∼90% of patients with advanced PCa have skeletal metastasis [4]. Bone metastasized PCa is virtually incurable and is associated with severe morbidity before death, these include bone pain, pathological fractures, nerve compression syndromes, and hypercalcemia [4]. Currently, the available treatment options for patients with metastatic disease are palliative. The prognosis/diagnosis of bone metastatic lesions is currently determined by imaging using isotope bone scanning, computed tomography (CT) scan, magnetic resonance imaging (MRI) scan, or bone biopsy. The identification of prostate biopsy or serum based biomarker(s) for predicting the susceptibility of men to develop metastasis will potentially better discriminate the more aggressive metastatic forms of the disease and thus provide better treatment and clinical management opportunities for the disease.

Over the years the utility of PSA as a biomarker for prostate cancer has been controversial with respect to its inability to distinguish indolent from aggressive forms of the disease [5], [6]. PSA is also associated with high rates of false-positive and false-negative test results, as levels may be elevated in non-cancer conditions of the prostate, including benign prostatic hyperplasia (BPH) and prostatitis [7]–[10]. Recently the U.S. Preventive Services Task Force (USPSTF) recommended against PSA-based screening for PCa in all age groups stating that the benefits do not outweigh the harms of screening and treatment [11]. This inability to accurately predict the aggressiveness of prostate cancer based solely on standard clinicopathologic features clearly underscores the need to explore the ability of tumor-based biomarkers to enhance outcome prediction at biopsy and to understand the molecular basis of prostate cancer metastasis. Therefore, additional biomarkers are urgently needed to improve the diagnostic specificity of PSA and predict the potential of disease progression.

To better understand the molecular mechanisms of prostate cancer metastasis, it is crucial to identify the markers that are associated with metastases. Proteomics has proved to be a useful and successful approach in screening tumor and metastases related protein markers [12]. There are several proteomics technologies that have been applied in screening and identifying potential cancer markers. The ‘isobaric Tags for Relative and Absolute Quantitation’ (iTRAQ) platform has the advantages of being relatively high throughput and it can be multiplexed to provide information on peptide/protein quantitation and identification, as reported in previous studies [13], [14]. In this approach multiple samples from different proteomes are reduced, alkylated and proteolytically digested to generate peptides. The peptides are labeled with a set of iTRAQ reagents (in a 4 or 8-plex format), pooled and fractionated by strong cation exchange (SCX). The fractions are then analyzed by liquid chromatography tandem mass spectrometry (LC-MS/MS), with the resultant mass spectra providing sequence information (from the peptide fragments), and relative quantification (from the reporter group ions).

In an effort to identify novel proteins that are associated with the metastatic progression of human prostate cancer, we have performed a 4-plex iTRAQ analysis using two syngeneic prostate cancer cells lines PC3-N2 and PC3-ML2 which have different metastatic potentials. PC3-N2 is lowly metastatic while PC3-ML2 is highly metastatic. Following comparative quantitative data analysis, a number of candidates were found to be significantly differentially expressed in PC3-ML2 cells compared to PC3-N2 cells. Two of the candidates identified as being significantly up-regulated in PC3-ML2 are plectin and vimentin. These proteins were further investigated by Western blot and siRNA knockdown. The expression levels correlated to immunohistochemistry images from normal and adenocarcinoma prostate tissue samples. The differentially regulated proteins identified in the study, including plectin and vimentin, are discussed in the context of their significance to prostate cancer progression.

## Experimental Procedures

### Materials

Dulbecco's modified Eagle media (DMEM) and fetal bovine serum (FBS), antibiotics-antimycotics, 4–12% NuPAGE® Bis-Tris gels and 2×Laemmli buffer were from Invitrogen (Carlsbad, CA). Complete™ protease inhibitors were purchased from Roche Applied Sciences (Indianapolis, IN), sequencing grade trypsin was from Promega (Madison, WI), and Immobilon-FL PDVF membrane was from Millipore (Billerica, MA). BCA protein concentration assay kit and the protein-free blocking buffer were from Thermo Scientific (Rockford, IL). The primary antibodies for vimentin (ab20346), plectin (ab83497) were from Abcam (Boston, MA), and plectin (sc-7572), GAPDH (sc-25778), pERK 1/2 (Thr 202/Tyr 204) sc-16982, ERK 1/2 (MK1) sc-135900, and CDC2 p34 (sc 53) were from Santa Cruz Biotechnology Inc. (Santa Cruz, CA). CDC2 (Cat #9112) antibody was from Cell Signaling Technology. IRDye conjugated secondary antibodies (Goat anti-mouse IR680 Cat. # 926-3220, Goat anti rabbit IR800CW Cat. # 926-32211, Goat anti rabbit IR680 (Cat. # 926-32221), Donkey anti-goat IR680 (Cat. # 926-32224), Goat anti- mouse IR800CW (Cat. # 926-3220) were from Li-COR Biosciences (Lincoln, PA) and Alexa Fluor secondary antibodies for immunofluorescence confocal microscopy were from Invitrogen (Carlsbad, CA). Control siRNA-A (sc-37007), vimentin (h) (sc-29522) and plectin siRNA (h) (sc-29453) were from Santa Cruz Biotechnology Inc. (Santa Cruz, CA).

### Cell Lines and Cell Culture

Parental PC3 were originally obtained from a skeletal metastasis in a patient with primary prostate adenocarcinoma. The two PC3-N2 and PC3-ML2 sublines were kindly donated to us by Dr. Stearns' group at Drexel University and were developed as described by Wang and Stearn [15]. The cell lines were developed on the basis of their invasiveness *in vitro* and metastatic potential *in vivo*. Both cells were tumorigenic when injected subcutaneously into SCID mice. However, N2 cells were unable to migrate through a matrigel-coated membrane *in vitro* as well as induce metastases in SCID mice, whereas ML2 cells were highly invasive *in vitro* and induced skeletal metastases in more than 80% of cases [15]–[17]. PC3-N2 and PC3-ML2 cells were cultured in DMEM medium supplemented with 10% FBS and 1% antibiotics at 37°C with 5% CO_2_. Cells then were dissociated from the plastic surface using 5 mM EDTA in PBS. The non-enzyme dissociation buffer preserves cell surface molecules and cell viability.

### Protein Extraction Digestion and ITRAQ Labeling

For the total cell lysate experiments PC3-N2 and PC3-ML2 cells were cultured in complete growth medium up to 80% confluence. Cells detached using 5 mM EDTA in PBS and washed with PBS and the pellet resuspended in 160 µl dissolution buffer containing 100 mM NH_4_HC0_3_ and TFE (1∶1 v/v). The samples were sonicated for 20 seconds three times and incubated at 60°C for 1 h. The lysates were centrifuged to remove cell debris and unbroken cells before collecting the supernatant. The protein concentration was determined by BCA assay and normalized for each sample. 100 µg aliquots of the samples were dried in a SpeedVac and subjected to trypsin digestion and peptide labeling with iTRAQ reagents according to the manufacturer's instructions (iTRAQ Reagents Multiplex Kit; ABSciex, Foster City, CA). Briefly, 100 µg of proteins were vacuum-dried and resuspended in 20 µl of dissolution buffer and 1 µl of denaturant at RT. Samples were reduced, alkylated and trypsinized with 5 µg modified sequencing grade trypsin (Promega, Madison, WI, USA) for 18 h at 37°C. Trypsin digested samples were labeled with four different iTRAQ reagents dissolved in 70 µl of ethanol at room temperature for 1 h. Reactions were quenched with 10 mM glycine. The samples were as follows: PC3-N2 cells samples with 114 and 115 tags and PC3-ML2 samples with 116 and 117 tags. This strategy provides internal technical replicates for the two types of samples. All the four labeled samples were pooled, vacuum-dried and fractionated utilizing a strong cation exchange (SCX) column.

### 2D-LC Separations

In the first dimension, SCX separations were performed on a passivated Waters 600E HPLC system, using a 4. ×250 mm polysulfoethyl aspartamide column (PolyLC, Columbia, MD) at a flow rate of 1 ml/min. Buffer A contained 10 mM ammonium formate, pH 2.7, in 20% acetonitrile/80% water. Buffer B contained 666 mM ammonium formate, pH 2.7, in 20% acetonitrile/80% water. The gradient was Buffer A at 100% (0–22 minutes following sample injection), 0%→40% Buffer B (16–48 min), 40%→100% Buffer B (48–49 min), then isocratic 100% Buffer B (49–56 min), then at 56 min switched back to 100% A to re-equilibrate for the next injection. The first 26 ml of eluant (containing all flow-through fractions) was combined into one fraction, and then 14 additional 2-ml fractions were collected. All 15 of these SCX fractions were dried down completely to reduce volume and to remove the volatile ammonium formate salts, then resuspended in 9 µl of 2% (v/v) acetonitrile, 0.1% (v/v) trifluoroacetic acid and filtered prior to reverse phase C18 nanoflow-LC separation. For the 2nd dimension separation by reverse phase nanoflow LC, each SCX fraction was automatically injected onto a Chromolith CapRod column (150×0.1 mm, Merck) using a 5 µl injector loop on a Tempo LC MALDI Spotting system (ABI-MDS/Sciex). Buffer C was 2% acetonitrile, 0.1% trifluoroacetic acid, and Buffer D was 98% acetonitrile, 0.1% trifluoroacetic acid. The elution gradient was 95% C/5% D (2 µl per minute flow rate from 0–3 min, then 2.5 µl per minute from 3–8.1 min), 5% D→38% D (8.1–40 min), 38% D→80% D (41–44 min), 80% D→5% D (44–49 min) (initial conditions). Flow rate was 2.5 µl/min during the gradient, and an equal flow of MALDI matrix solution was added post-column (7 mg/ml recrystallized CHCA (α-cyano-hydroxycinnamic acid), 2 mg/ml ammonium phosphate, 0.1% trifluoroacetic acid, 80% acetonitrile). The combined eluant was automatically spotted onto a stainless steel MALDI target plate every 6 seconds (0.6 µl per spot), for a total of 370 spots per original SCX fraction.

### Mass Spectrometry Analysis

The sample spots were dried and calibrant spots (ABI 4700 Mix) are added to each plate manually. The MALDI target plates (15) were analyzed in a data dependent manner on an ABI 5800 MALDI TOF-TOF after calibration. Mass spectrometry (MS) spectra were acquired from each sample spot using the newly updated default calibration, using 500 laser shots per spot. A plate-wide interpretation was automatically performed to choose the highest peak of each observed m/z value for subsequent tandem MS/MS analysis. Up to 2500 laser shots at laser power 4200, with collision induced dissociation (CID) gas air at 1.2 to 1.3×10^−6^ Torr were accumulated for each MS/MS spectrum. After the MS and MS/MS acquisition, spectra from all 15 plates in the sample were used for protein identification and quantitation using the Paragon algorithm as implemented in Protein Pilot 4.0 software (ABI-Sciex) [18]. The spectra were searched against the human subset (plus common contaminants) of the NCBI non redundant database concatenated with a reversed "decoy" version the same database using the latest of these FASTA databases, obtained from http://www.ncbi.nlm.nih.gov/sites/entrez?db=Taxonomy &cmd = search&term = . (Ref Seq Human database 20120204 sequences containing 29766 Protein Sequences, plus 389 common lab contaminants. The Protein Pilot search parameters were: Methyl methanethiosulfonate (MMTS) alkylated cysteine residues, 4-plex iTRAQ modification of lysine and N-terminus with an identification focus on biological modifications and amino acid substitutions using the thorough search effort. The False Discovery Rate (FDR) estimation was determined by simultaneously searching the concatenated decoy database [19].

### Pathway Analysis

Data generated from proteomic mass spectrometric analysis were analyzed using Ingenuity Pathway Analysis (IPA; Ingenuity System, Redwood city, CA, www.ingenuity.com). Ingenuity Knowledge Base tool was used to identify biological function and canonical pathways that include and involve the differentially regulated proteins. IPA is a regularly updated database which uses the current knowledge available on genes, proteins, normal cellular and pathological processes, signaling and metabolic pathways, needed for pathway construction.

### Protein Preparation for Validation

The PC3-N2 and PC3-ML2 cells were cultured to 80% confluence and detached using 5 mM EDTA in PBS. For protein extraction, cells were washed twice with PBS and lysed in modified RIPA buffer [50 mM Tris-HCl (pH 7.4), 1 mM EDTA, 150 mM NaCl, 1% Triton X-100, 0.25% sodium deoxycholate] containing a protease inhibitor cocktail (Complete, Mini, Roche) at 4°C for 15 min. Samples were sonicated for 1 min and centrifuged at 14000 g for 15 min at 4°C, and the supernatant was collected. Equal amounts of protein (BCA Protein Assay Kit, Pierce) from each sample were separated on a 4–12% Tris-glycine sodium dodecyl sulfate-polyacrylamide gel by electrophoresis (SDS-PAGE), followed by Western blot transfer to PVDF. For the Western analysis, electrophoresed proteins were transferred onto PVDF membrane, blocked with Odyssey Blocking Buffer (Rockland Immunochemicals, Gilbertsville, PA), probed with the primary antibodies overnight at 4° C. After washing, the membranes were incubated with the respective IRDye conjugated secondary antibodies and visualized using an Odyssey infrared imaging system (Li-COR Biosciences, Lincoln, Nebraska).

### Transfection and Silencing of Plectin and Vimentin by siRNA

SiRNA duplexes were used to silence plectin and vimentin expression. A non-targeting 20–25 siRNA duplex was used as the negative control. SiRNA duplexes were transfected into the PC3 cell lines with siRNA transfection reagent according to the manufacturer's instructions Santa Cruz Biotechnology Inc. (Santa Cruz, CA). After transfection for 72 h, cells were subjected to Western blot and immunofluorescence analysis to detect the efficiency of vimentin and plectin knockdown and to determine the effects of the knockdown on the migration and invasive properties of the cells.

### Confocal Microscopy Analysis

PC3-N2 and PC3-ML2 cells were seeded onto 6-well plates containing glass cover slips and cultured in 10% FBS/DMEM for 2 days. Cells were washed with ice-cold PBS, fixed with ice-cold methanol for 5 min. Subsequently, the fixed cells were washed with PBS and stained with primary antibodies against plectin, vimentin, and CDK2 overnight at 4°C. The primary antibodies were removed and the cells were washed with ice cold PBS before staining with Alexa Fluor conjugated secondary antibodies for 1 hour at room temperature. TOPRO stain was included with the secondary antibodies for nuclear staining. The secondary antibodies and nuclear stains were washed away and the cover slips mounted to the slides with Vector Shield medium containing DAPI stain (Vector Labs, Burlingame, CA), and sealed with nail polish. Fluorescent images were examined and captured using a confocal microscope (Carl Zeiss, Thornwood, NY).

### Cell Proliferation Assay

PC3-N2 and PC3-ML2 cells in 24-well dishes at a density of 2 ×10^4^ cells and transfected with non-targeting and plectin/vimentin specific siRNA at a final concentration of 20 nM. Both attached and floating cells were collected after low speed centrifugation and trypsination. The cells were stained with Trypan blue, and the number of Trypan blue-positive cells were counted on days 2, 4, 6 post transfection.

### Scratch Assay/Wound Healing Invasion Assay

The migration ability of the two cell lines was evaluated by a wound healing assay. Briefly, PC3-N2 and PC3-ML2 cells were plated in 6-well dishes at a density of 2 ×10^5^ cells and transfected with non-targeting and plectin/vimentin specific siRNA at a final concentration of 20 nM for 72 hours. An artificial wound was carefully created at 0 h using a 200 µl pipette tip to scratch the confluent cell monolayer in triplicate for each condition. The cells were washed with PBS and cultured in 10% FBS-DMEM at 5% CO2 and 37°C. A photomicrograph was taken immediately after scratching (time 0 h) and 24 h later under 10×objective lens using an inverted Zeiss AxioObserver.A1 phase-contrast microscope (Zeiss, Oberkochen, Germany). The migration distances in the triplicate wells were measured and the mean and standard error values compared between the control and the plectin and vimentin knockdown cells.

### Trans-well Migration Assays

To investigate the invasiveness of the two cell lines, a membrane invasion culture system was used as follows. Cells which pretreated with control siRNA or vimentin and plectin siRNAs were starved overnight in DMEM media with 0.5% FBS, then were trypsinized and resuspended into DMEM containing 0.5% bovine serum albumin. Cells (1×10^5^) were added to the top chambers of 24-well transwell plates (Corning, 8 µm pore size), and DMEM with 10% FBS and LPS was added to the bottom chambers. Lower chambers were filled with culture medium containing either 0.5% FBS as a control or containing 10% LPS as an attractant. The plates were incubated at 37°C for 24 hours. At the end of the incubation period, the upper surface of the membrane was wiped with a cotton-tip applicator to remove non-migratory cells. Cells that migrated to bottom side of membrane were fixed and stained with hematoxylin to visualize nuclei. The cells were quantified by counting five high powered fields under 100×magnification, and the means for each chamber were determined in triplicate. Percent invasion was represented as mean number of cells migrating through the membrane for the plectin and vimentin silenced cells relative to the non-targeting siRNA.

### Correlation of Plectin and Vimentin Expression Using Human Proteome Atlas

The Human Protein Atlas database displays expression and localization patterns of proteins in a large portion of human tissues and organs with an emphasis on strategies for validating immunohistochemistry based protein expression patterns for cancer research projects. The ultimate objectives for the project include the creation of an information database of protein expression patterns in normal human tissues, in cells and in cancer, and utilizing generated antibodies and protein expression data as tools to identify clinically useful biomarkers [20], [21].The database contains high resolution images annotated by a certified pathologists. The Human Protein Atlas was used to screen for the expression of plectin and vimentin focusing on immunohistochemistry of normal and cancerous prostate tissue. These comparative analyses in combination with the data generated in the present study will provide the basis of future hypothesis driven investigations of the role of these proteins in prostate cancer disease progression.

### Statistical Analysis

Each of these experiments was conducted in triplicate. Data are expressed as mean ± standard error of mean (S.E.M.). Statistical analysis was performed using SPSS 13.0 statistical software (SPSS, Inc. Chicago, IL, USA). *P*<0.05 was considered significant.

## Results

### Identification of Differentially Expressed Proteins in PC3-N2 and PC3-ML2 Cell Lines Using iTRAQ Labeling and 2D LC-MS/MS

We have analyzed the proteome of syngeneic prostate cancer cell PC3-N2 and PC3-ML2 by 2D LC-MS/MS using iTRAQ, to generate data of differentially expressed proteins. The overall experimental workflow is shown in Supplementary Figure S1. A total of 1756 non-redundant proteins were repeatedly identified by duplicate iTRAQ labeling and 2D LC-MS/MS analyses, 95% of which were identified with >2 peptide matches. The false discovery rate (FDR) for proteins identification based on searching against a reversed database was 1%. The detailed information including information of peptide sequences, protein quantification date, average iTRAQ ratio, and distinct and common peptides with a group of proteins for these identified proteins is shown in Table S1. To identify differentially expressed proteins in PC3-N2 and PC3-ML2 that may be related to invasion and metastasis, protein expression profiles between the two cell lines were compared. The proteins that met the following criteria were confidently considered as differentially expressed proteins: (i) proteins were repeatedly identified by the duplicate experiments; (ii) proteins were identified based on≥2 peptides; (iii) proteins showed an averaged ratio-fold change≥1.5 or≤0.667 in the duplicate experiments between the two cell lines (*t* test, *p*<0.05). Using these criteria, 130 proteins were found to be differentially expressed in PC3-N2 and PC3-ML2 cells. Of these proteins, 62 were found to be up-regulated while 68 were down-regulated. The names of these 130 proteins and their expression levels are shown in Table S1. Some of the MS/MS spectra used for the identification and quantitation of PLEC1 and VIM with significantly high ratio-fold changes in PC3-ML2 cells compared to PC3-N2 lines are shown in Figures S 2A and S 2B. The MS/MS spectrum for GAPDH which did not have fold changes in the two cell lines is shown in Figure S 2C. EphA2 is an example of a protein that is down-regulated in PC3-ML2 vs PC3-N2 and this is shown in Figure S 2D. A high concordance was observed between the two reporter ion intensities for a given peptide for virtually all peptides identified. The average fold-change for a protein was obtained by using all the peptides identifying that protein. Therefore our protein identifications and quantitation are consistent and of high confidence. This subset of 130 differentially regulated proteins was used for all subsequent bioinformatics analysis, biological annotations and interpretation.

### Pathway Analysis

**Figure 1:**
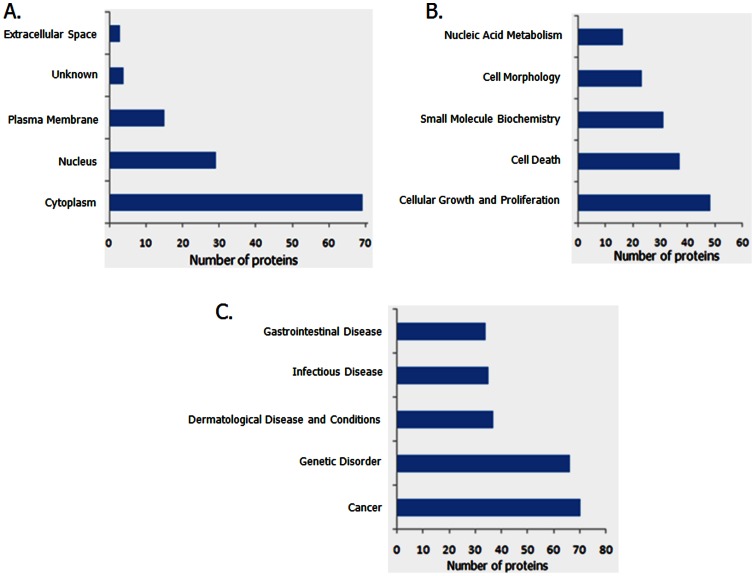
Subcellular location, molecular functions and disease classifications of differentially regulated proteins. Ingenuity knowledge base analysis was used to determine the sub-cellular localization, molecular and biological functions and disease processes that are associated with the proteins that are differentially regulated between PC3-N2 and PC3-ML2 cells. The numbers of proteins are shown in the x-axis and in Tables S2 and S3.

Using the Ingenuity software the differentially regulated proteins were assigned a subcellular localization, molecular and cellular functions, and their possible involvement in diseases and disorders. Figures 1A, B and C display their sub-cellular localization, their cellular and molecular functions, as well as the main diseases and disorders that they represent respectively. Tables S2 and S3 summarize the different functional categories and their p values. Our results indicate that the differentially regulated proteins between the two syngeneic prostate cancer cell lines are mainly involved in cancer, dermatological disease, genetic disorders, gastrointestinal disease, and immunological disease. Using the IPA knowledge base tool, we have identified the top networks and canonical pathways which suggest the main possible functions of proteins that are differentially expressed in the two cell lines. As indicated in Table S4 and Figure S3, the top network is cell morphology, connective tissue development and function, and cellular movement.

### Validation of Differentially Expressed Proteins Identified by Proteomics

**Figure 2:**
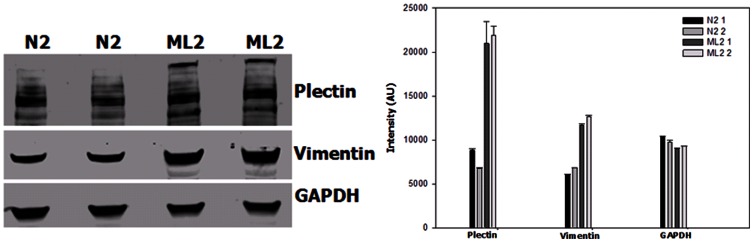
Validation of differentially upregulation of plectin and vimentin in PC3-ML2 cells. Total cell lysates (40 µg) of N2 and ML2 cells were subjected to SDS-PAGE. The separated proteins were analyzed by Western blot analysis to detect plectin and vimentin as described. GAPDH detection was included as a loading control.

**Figure 3:**
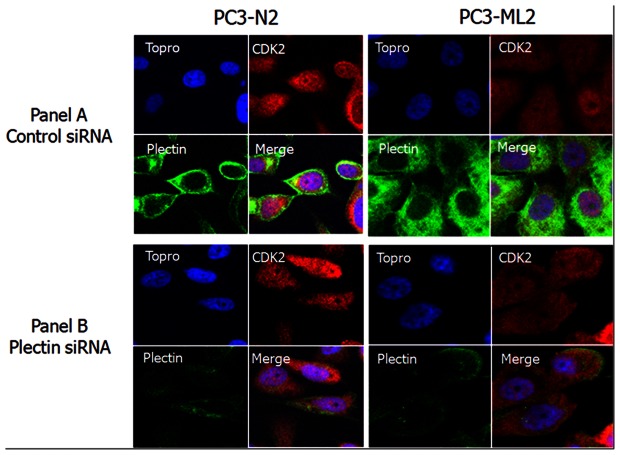
Confocal microscopy of PC3-N2 and PC3-ML2 cells for visualization of Plectin and CDK2. Cells were treated with control siRNA or Plectin gene specific siRNA for 3 days and then fixed, incubated with rabbit polyclonal anti-plectin and mouse monoclonal anti-CDK2 primary antibodies before "staining" with Alexa Fluor conjugated donkey anti-rabbit secondary antibodies (green), goat anti-mouse secondary antibodies (red) and the nuclear stain TOPRO (blue). Panel A shows the images for control siRNA transfected cells and Panel B shows the Plectin siRNA transfected cells.

Among the 130 proteins with altered expression, we focused on two proteins plectin and vimentin, which were very significantly upregulated in PC3-ML2 vs PC3-N2. In addition, their expression levels have not been well studied in prostate cancer. Western blotting was performed to detect the expressional levels of the two proteins in triplicate experiments. As shown in Figure 2, PLEC1 and VIM expression levels are significantly increased in PC3-ML2 vs PC3-N2, which is consistent with MS analysis data. The relative expression levels of plectin normalized and vimentin with GAPDH level are indicated adjacent to the Western blotting image. In complementary analyses, immunofluorescence assays verify efficient knocking down of the plectin and further validate the mass spectrometry data as shown in Figure 3 panel A where the intensity of plectin staining is shown to be significantly highly expressed in PC3-ML2 compared to PC3-N2. The same was observed for the vimentin knockdown cells (Figure S4)

### Plectin and Vimentin Knockdown Inhibits Proliferation of PC3-ML2 Cell Lines

**Figure 4:**
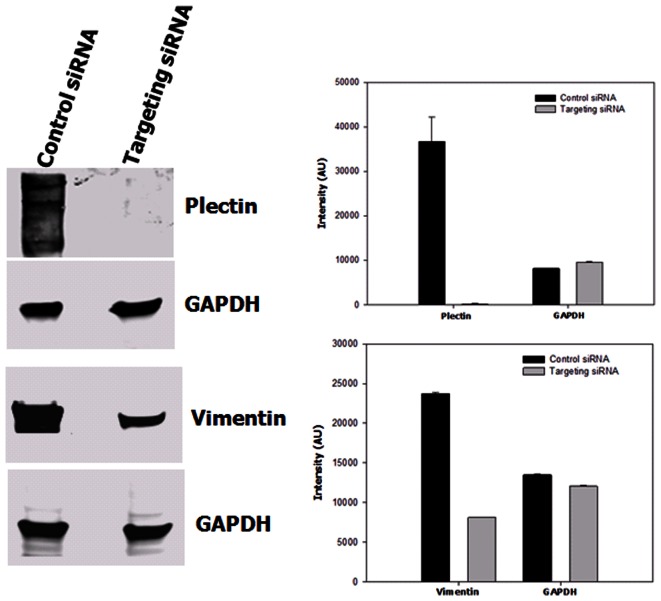
Validation of siRNA knockdown of plectin and vimentin in PC3-ML2 cells. Cells were treated with control siRNA, plectin or vimentin gene specific siRNA. Total cell lysates (40 µg) of ML2 cells were subjected to SDS-PAGE. The separated proteins were analyzed by Western blot analysis to detect plectin and vimentin as described. GAPDH detection was included as a loading control.

**Figure 5:**
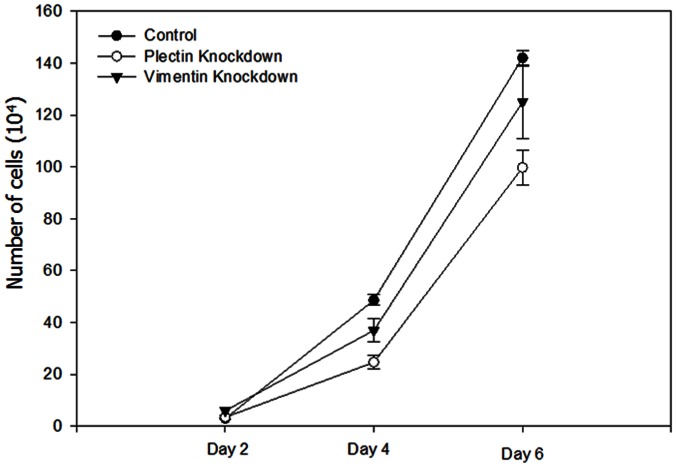
Suppression of plectin and vimentin inhibits the proliferation of PC3-ML2 cells. Cells were transfected with control, plectin and vimentin siRNA. Cells were cultured and counted every two days for at total of 6 days. There is a significant difference in the proliferation rates between control siRNA compared with plectin and vimentin siRNA, P<0.05.

We examined whether plectin and vimentin contribute to cell proliferation in prostate cancer using the RNAi technique. After introduction of plectin and vimentin siRNA into PC3-ML2 cells for 72 hr, suppression of plectin and vimentin were confirmed by Western blotting, while the control non-targeting siRNA had no significant effect on endogenous plectin and vimentin expression (Figure 4). The expression of plectin and vimentin were reduced by 72% and 99.9% respectively in comparison to the controls (*p*<0.05). The relative expression of GAPDH in the controls and plectin/vimentin targeted siRNA knockdowns are identical. The proliferation of PC3-ML2 cells was examined by cell counting 2–6 days after siRNA treatment. Our data show that cell proliferation was significantly reduced by both plectin and vimentin knockdown (Figure 5). Data from complementary assays show no significant differences in the viability of both cell lines (Figure S5).

### Knockdown of Plectin and Vimentin Reduces Invasion and Migration Capacity of PC3-ML2 Cells

Cell migration is crucial for the invasion of cancer cells. Plectin interlinks intermediate filaments with microtubules and microfilaments and anchors intermediate filaments to desmosomes or hemidesmosomes. It could be involved in tumor cell migration and invasion which has been shown in several cancers [22]–[25]. Vimentin is required to maintain the architecture of the cytoplasm in cells. Vimentin intermediate filaments, in addition to their potential interactions with microfilaments and microtubules, participate in many other specialized cell functions [26]. During tumor metastasis, cancer cells undergo epithelial–mesenchymal transition (EMT) in which epithelial tumor cells in the primary cancer site are converted into aggressive and metastatic tumor cells. One of the main characteristics of EMT is the combination of loss of cell–cell contact, reduced E-cadherin expression, and enhanced expression of mesenchymal markers such as vimentin [27]. The mesenchymal-like cells generated are transported to metastatic sites, where they may undergo mesenchymal–epithelial transition by regaining E-cadherin expression, allowing cell–cell adhesion and connecting adjacent cells to form new foci [28].

**Figure 6:**
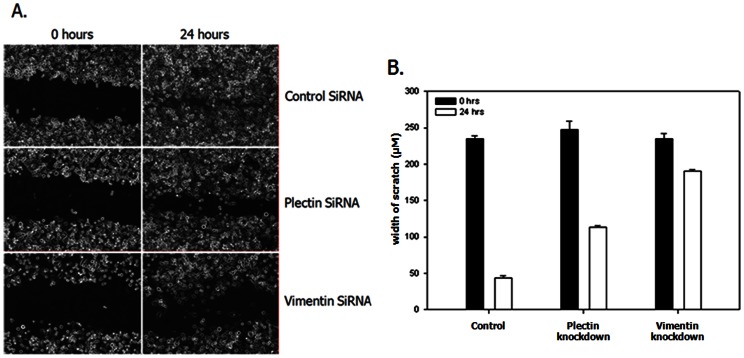
Plectin and vimentin suppression significantly reduces the migration ability of PC3-ML2 cells. A. Confluent monolayer PC3-ML2 cells were scratched with a pipette tip and migration toward the wounded area was observed and measured. B. There is a significant difference in the migration rates between control siRNA compared with plectin and vimentin siRNA, P<0.05.

To determine whether plectin and vimentin are involved invasion and migration and thus potentially prostate cancer metastasis, we transfected PC3-ML2 with siRNA targeting the two genes. The transfected cells were used in scratch/wound healing assays to determine the consequences of the gene knockdowns. In these experiments the extent of wound closure is a direct measure of cell motility or migration capacity. In the cells transfected with the control non-targeting siRNA the wound closure was almost complete within 24 hours of transfection in the PC3-ML2 cells. In contrast the wound was still present in PC3-ML2 cells into which plectin and vimentin siRNA had been introduced (Figure 6). Therefore cell migration was significantly inhibited by both plectin and vimentin knockdown cells compared to the non-targeted siRNA controls.

**Figure 7:**
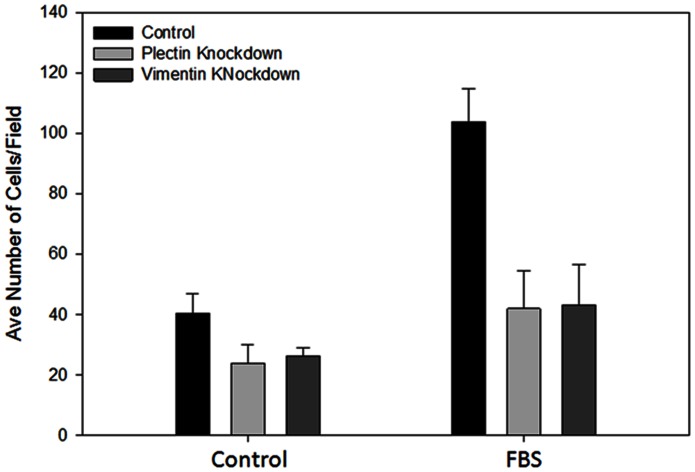
Suppression of plectin and vimentin significantly reduces the invasion ability of PC3-ML2 cells. Cells were transfected with control, plectin and vimentin siRNA and allowed to invade using a transwell migration assay. The invading cells were stained and three randomly selected fields were counted. The number of invading cells was significantly lower in the plectin and vimentin siRNA compared to the control siRNA transfected cells, P<0.05.

We further examined the effect of plectin and vimentin knockdown on PC3-ML2 cell invasion using the transwell migration assay. Control cells and plectin and vimentin knockdown cells passing through the insert membrane was significantly reduced in plectin and vimentin knockdown invasive cells as compared with the control non-knockdown cells. Percent invasion of plectin and vimentin knockdown cells was significantly lower than that of control siRNA-treated cells (Figure 7). We therefore conclude that a reduction in the levels of both plectin and vimentin produced by the cells resulted in suppressed cell invasion in the prostate cancer cell line. Taken together, these results demonstrate that knockdown of plectin and vimentin decreases the motility of PC3 cell lines thus, supporting previous reports that down-regulation of these proteins is involved in invasion and migration important initial steps in cancer metastasis. This is further supported by the lack of plectin expression in the benign non-tumorigenic epithelial RWPE-1 prostate cancer cell line (Figure S6).

### Signaling Pathways Regulation in Plectin and Vimentin Knockdown Cells

**Figure 8:**
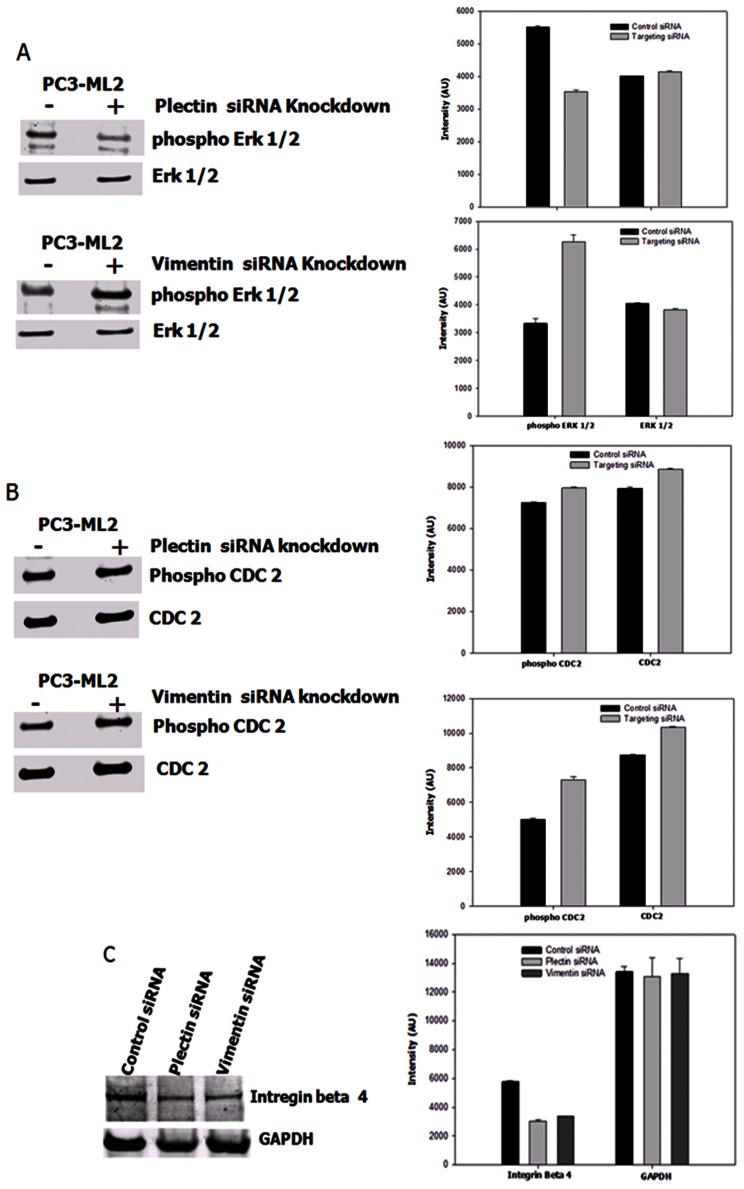
Plectin and vimentin suppression modulate signal transduction events and pathways knockdown PC3-ML2 cells. **A and B.** The relative expression levels of each protein were compared to the amounts in the control siRNA. The relative expression levels of each protein were compared to the amounts in the control siRNA. There is a decrease of p-Erk 1/2 in the plectin knockdown cells compared to the controls, and an increase in p-Erk1/2 in the vimentin knockdown cells compared to the controls. C. Integrin beta 4 is suppressed in both the plectin and vimentin transfected cells.

Plectin plays a crucial role in maintaining the integrity of the cytoskeleton by interlinking intermediate filaments with other cytoskeletal network systems, and anchoring them to the plasma membrane. It also serves as a scaffolding platform for signaling cascades. Our Ingenuity Pathway Analyses identified the PI3K complex and ERK as central nodes in the top network of the analyses. Akt and Erk1/2 are two major signaling pathways in regulating cell proliferation, migration and survival. Downstream effectors of Akt are involved in survival, growth and metabolic-related pathways. Upregulation of Erk 1/2 kinases has been shown to play a role in tumor invasion by inducing EMT [29], [30], or by promoting the degradation of extracellular matrix proteins through induction of MMPs [31]. The mechanism of the inhibition of migration and invasion in plectin knockdown cells has been controversial. It has been previously postulated that basal phosphorylation of Erk 1/2 kinases was significantly elevated in plectin−/− keratinocytes and was the cause of the faster migration of plectin-deficient cells compared to the plectin+/+ cells [32]. This is contrary to the recent results from Katada et al showing a significant reduction in phosphorylated Erk 1/2 in plectin knockdown HNSCC cells as compared to controls [25]. Ding et al. have also reported that knockdown of plectin with RNA interference (RNAi) reduces the activation of Erk1/2 in HEK293 cells [33]. We therefore investigated Erk 1/2 activities in plectin and vimentin knockdown PC3-ML2 cells. Western blot analyses show that there is no difference in total Erk 1/2 level between plectin siRNA knockdown and control siRNA-treated PC3-ML2 cells (Figure 8A). However, there is a 1.6 fold reduction in the amount of phosphorylated Erk 1/2 in the knockdown cells compared to the controls (Figure 8A). These results support the work of Katada et al., and suggest that the inhibition of migration and invasion in plectin knockdown cells may be associated with down regulation of Erk 1/2 kinase activities and that the effect on migration in plectin-deficient cells depends on the different response of Erk 1/2 activity to the alteration of plectin expression in each cell line [25]. In the case of vimentin, the reverse is observed and the relative amount of phosphorylated Erk 1/2 is increased 1.82 fold in vimentin knockdown PC3-ML2 cells compared to the controls. To our knowledge, this report describes for the first time that an increase in the kinase activity of Erk 1/2 in prostate cancer cell lines is associated with vimentin knockdown and the observed decrease in invasion and migration potential. In subsequent experiments we show that there are no differences in the expression levels of both total and phosphorylated Akt in both PC3-ML2 control, and plectin or vimentin knockdown cells (data not shown). Plectin has a unique phosphorylation site for protein kinase Cdk1 (CDC2) [34]. In our current studies, the expression of Cdk1 in N2 and the ML2 cells is the same and there are no significant differences in the amounts of phosphorylated Cdk1 (CDC2) after siRNA knockdown of both plectin and vimentin (Figure 8B). However, the amount of integrin β4 which has been shown to interact with plectin is down regulated in both plectin and vimentin knockdown cells by approximately 47% (Figure 8C). This would suggest that the interaction between these two proteins with integrin β4 may play a role in the invasion and metastatic potentials of these cell lines.

### Plectin and Vimentin are over Expressed in Human Prostate Adenocarcinoma

**Figure 9:**
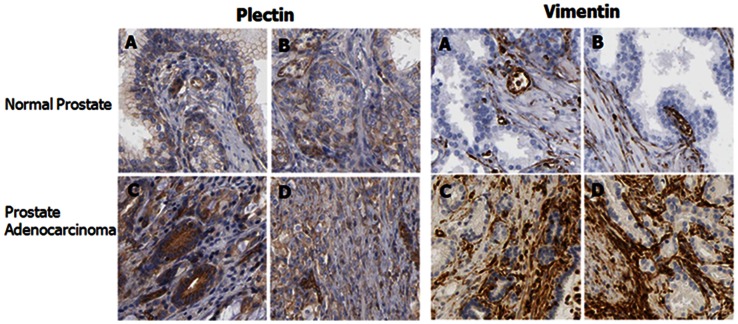
Representative images showing the immunoexpression of plectin and vimentin in prostatic tissue. The Human Protein Atlas was used to screen for the expression of plectin and vimentin focusing on immunohistochemistry of normal (upper panels A and B) and cancerous prostate tissue (lower panels C and D). Immuno-expression of both plectin and vimentin are higher in the malignant adenocarcinoma tissues compared to the normal tissues.

We have used the Human Protein Atlas database to compare the tissue expression profiles for plectin and vimentin by immunohistochemistry [20]–[21]. The database contains high resolution images of normal and adenocarcinoma prostate tissues annotated by a certified pathologists but it does not have images for distant metastasis tissues. The database includes images of various cell lines and the PC3 parent cell line shows moderate staining for both plectin and vimentin (data not shown). The expression of plectin is weak in normal tissues (Panels A (tissue ID # 2053) and B (tissue ID # 2098)) but is in significantly increased to moderate staining in high grade adenocarcinoma tissues (Panels C (tissue ID # 3191) and D (tissue ID # 472)) (Figure 9). In both conditions the protein is localized in both the cytoplasm and membranous structures in the cells. For vimentin, there is negative staining of the protein in normal tissues (Panels A (tissue ID # 2053) and B (tissue ID # 2098)) but there is strong staining for the protein in both the cytoplasm and membranous structures in the cells from medium grade adenocarcinoma tumor tissues (Panels C (tissue ID # 613) and D (tissue ID # 244)) (Figure 9). These IHC data correlate the upregulation of these two proteins with disease progression and is consistent with our current study which shows an increase in the expression of these proteins with an increase in metastatic potential.

## Discussion

Cancer metastasis is a very complicated process that involves a coordinated expression of multiple genes and proteomics can be used to study these global changes in protein expression in the disease processes. We have previously used glycan metabolic labeling with mass spectrometry analysis to identify and characterize metastasis-associated cell surface sialoglycoproteins in the PC3-N2/PC3-Ml2 syngeneic cell lines [35]. In the current study, we have determined the global expression of proteins in syngeneic prostate cancer cell lines with different metastatic potentials to determine their potential roles in metastasis using iTRAQ labeling and MUDPIT-LC-MALDI-MS/MS. These proteins could potentially serve as biomarkers for cancer diagnosis, prognosis, and therapeutic targets in prostate cancer.

Among these proteins, plectin and vimentin were significantly overexpressed in PC3-ML2 as compared to PC3-N2. Further functional analysis show that a decrease in the expression of plectin and vimentin suppresses the proliferation, migration and invasion of PC3-ML2 cells. Since both proteins have immunohistochemistry images available at the *Human Protein Atlas*, we systematically screened the available images for both normal prostate tissue and prostate cancer tissue. For both proteins there is a trend towards an increase in the intensity of staining in tumor tissue as compared to the normal prostate tissues. Plectin has been shown to interact with the integrin β4, a receptor for laminins, which is a major component of the epidermal basement membrane [36]. Integrins are a large family of heterodimeric transmembrane glycoproteins and function as cell-matrix, cell-cell adhesion receptor molecules and regulate complex ion and molecular environment around the cells [37]. Integrin β4 has been reported to associate with cell migration and invasion [38]. Furthermore, its high expression is associated with poor prognosis in a variety of human cancers [39].Thus, overexpression of plectin might contribute to cell migration and invasion in prostate cancer cells through its association with integrin β4.

Vimentin intermediate filaments, in addition to their potential interactions with microfilaments and microtubules, participate in many other specialized cell functions [26], [40]. Overexpression of vimentin has been associated with enhanced metastatic potential in breast amongst other malignancies [41], [42]. Vimentin is required to maintain the architecture of the cytoplasm, and aberrant vimentin expression during EMT is suggested to be an essential element for epithelial plasticity and tumor cell metastasis [43]. EMT trans-differentiation processes involve the conversion of adherent epithelial cells into individual migratory cells, leading to changes in cell phenotype into more loose mesenchymal-like cells, and promoting local invasion and metastatic dissemination of tumor cells [44], [45].

A recent study of isogenic prostate cancer cell lines using an 8-Plex ITRAQ comparative quantitative proteome analysis demonstrated the lack of vimentin expression in the androgen responsive LNCaP cells and almost identical expression levels in the androgen-independent PC-3 cells [46]. The study also demonstrates a significant overexpression of plectin in PC3 cells compared to LNCaP cells and a downregulation in PC-3M or PC-3M-LN4 compared to with the parental PC-3cells [47]. The methods by which these cell lines were generated and their invasive phenotypes are different from the cells that have been used in the present study. To our knowledge, our current study is the first to demonstrate that up-regulation of plectin and vimentin expression positively correlates with the invasion and metastasis of androgen-independent PCa cell lines.

The other proteins that are highly upregulated in PC3-ML2 include macrophage-capping protein (CapG), transgelin-2, galectin-1 and *N*-myc downstream regulated gene 1 (NDRG1) protein among others. Some of these proteins have been implicated in various aspects of tumorigenesis and these are hereby discussed. The CapG protein is important in the maintenance of cellular structure and morphology [48]–[55]. The overexpression of CapG in PC3-ML2 compared to PC3-N2 in this study would suggest that CapG can promote invasion and metastasis in prostate cancer. Transgelin (TAGLN) is ubiquitously expressed in smooth muscle tissues of normal adult vertebrates [55]. It has been recently demonstrated that transgelin prevents the binding of the androgen receptor co-activator to the androgen receptor resulting in the inhibition of androgen-stimulated cell growth in prostate cancer cells [56]. Transgelin has also been reported to play a role in tumor suppression in certain cells by interfering with ERK activation and AP-1 signaling to decrease the expression of MMP-9, which is extremely involved in invasiveness of cancer [57].NDRG1 (N-Myc downstream regulated gene 1), is localized in the nucleus, cytoplasm, cell membrane, and intracellular organelles [58]. Its regulation is rather complex, and is governed by hypoxia-inducible factor (HIF-1) and p53-dependent pathways [59], N-Myc, and probably many other factors at the transcriptional and translational levels, and through mRNA stability [60]. There are reports that NDRG1 is over-expressed in a variety of cancers, including lung, brain, melanoma, liver, prostate, breast, and renal cancer [61]–[62].Our data shows the upregulation of NDRG1 in the highly metastatic PC3-ML2 prostate cancer cell line and would suggest that the protein is up-regulated in metastatic disease. The other significantly upregulated proteins in PC3-ML2 cell lines compared to PC3-N2 include thymidine kinase 1 (TK1), oxysterols binding protein 2 (OSBP2), Annexin A5 (ANXA5), Glyoxalase I (GLO1), neurofilament light polypeptide (NEFL), prosaposin (PSAP), minichromosome maintenance protein 5 (MCM5) and the tumor metastatic process-associated protein 1(NME1). The potential association of these proteins with prostate cancer disease progression and management are discussed below. TK1 has been studied extensively, primarily as a diagnostic biomarker for a variety of cancer types and it is associated with proliferating cells and is primarily elevated during S phase [63], [64]. Oxysterols are oxygenated derivatives of cholesterol generated by enzymatic reactions mediated by cytochrome P450 family enzymes or by inflammation associated non-enzymatic reactions. Upregulation of OSBPs isoforms has been shown to occur in cholangiocarcinoma (CCA) metastasis [65], [66]. Annexin A5 is a Ca^2+^ -binding protein which is involved in membrane organization and dynamics. The invasion capacity of cancerous cells in oral carcinoma, prostate cancer has been shown to be partially regulated by annexin A5 [65], [66]. GLO1 plays a significant role as a detoxicant of methylglyoxal, a side-product of glycolysis. Cell lines with GLO1 amplification have also been shown to be more sensitive to inhibition of GLO1 by bromobenzylglutathione cyclopentyl diester (BBGC) and the gene may represent a useful target for therapy in cancers with GLO1 amplification [67]–[69]. In this study we report the significant upregulation of the neurofilament light polypeptide (NEFL) [70]. The significance of this observation with respect to chemotherapeutic response and survival in patients with prostate cancer needs to be addressed.

Prosaposin is a dual function glycoprotein that exists as the lysosomal precursor of sphingolipid activator proteins that function as essential co-factors for sphingolipid hydrolases. In the current study there is a significant upregulation of PSAP in the highly metastatic cell line compared to the non-metastatic cell line. This is in agreement with recently published data from patient samples [71]. A previous report demonstrated that prosaposin down-modulation decreases metastatic prostate cancer cell adhesion, migration, and invasion [72]. Prosaposin stimulates Tsp-1 and stromal p53 expression [73]. Further studies into the mechanism of PSAP stimulation of stromal p53 and Tsp-1 expression may provide therapeutic targets that could prevent the metastatic spread of human prostate tumors. Minichromosome maintenance (MCM) proteins are the core components of the DNA replication initiation machinery. It has been previously shown that MCM5 is overexpressed in prostate cancer and that raised MCM5 protein levels are an independent predictor of survival on multivariate analysis in patients treated with radical surgery [74]. Subsequent pilot studies have proposed the utility of MCM5 as a potential new biomarker for prostate cancer detection, but it is not yet clear whether the test will be able to specifically identify clinically significant cancers and this needs to be further investigated [75]. To date the role of NME1 in tumor development and progression remains uncertain. The abundance of this protein is reduced in some tumor cells of high metastatic potential but increased NME1 levels have been correlated with aggressive tumor features in neuroblastoma [76]. Therefore the protein may have distinct if not opposite roles in different tumors, and our results warrant further investigations on the role of NME1 in PCa progression.

Some of the significantly downregulated proteins in the highly metastatic PC3-ML3 cells compared to the non-metastatic PC3-N2 in the present study include moesin (MSN), the brain soluble protein 1 (BASP1), thyroid receptor interacting protein (TRIP13), annexin A2 (ANXA2) and ephrin type-A receptor 2 (EphA2). Moesin is a member of the Protein 4.1 superfamily and is believed to be involved in cell proliferation and growth. Immunohistochemical staining of moesin in prostatic adenocarcinoma show significantly reduced staining intensity in Stage 4 compared to Stage 2 PCa [77], and further studies on the protein may help elucidate its role in prostate cancer progression. BASP1 is a Wilms' tumor suppressor protein (WT1)-associated factor that can regulate WT1 transcriptional activity and it may be a potential target for prostate cancer therapy [78]. TRIP13 is a thyroid receptor interacting protein [79] whose gene shows copy number changes in 68% of 19 early stage NSCLC tumor samples [80]. TRIP13 has also been implicated as a marker of early disease related mortality in multiple myeloma as part of a 70-gene model. It is surprising to note that annexin A2 was been previously reported to be upregulated in various tumor types with a role in cell migration, invasion and adhesion [81], but is downregulated in PC3-ML2 compared to PC3-N2 in our current study.

Several studies have linked the overexpression of EphA2 to malignant progression [82], [83]. However, paradoxically, activation of EphA2 kinase on tumor cells can trigger signaling events that are more consistent with a tumor suppressor. These include inhibition of integrin signaling, Ras/ERK pathway, and Rac GTPase activation, which is correlated with inhibition of cell proliferation and migration [84]–[86]. Furthermore, EphA2 has also been shown to be a target gene for p53 family of proteins and it causes apoptosis when overexpressed [87]. Recent data supporting a tumor suppressor role of EphA2 include the demonstration that EphA2 is a key mediator of UV-induced apoptosis independent of p53 [88], and the dramatically increase insusceptibility to skin carcinogenesis in EphA2 KO mice [89]. Our current studies show a significant downregulation of EphA2 in the metastatic cells and future studies to determine how EphA2 may contribute to the progression of prostate cancer.

In summary, we have identified several proteins including plectin and vimentin that may act as markers for prostate cancer disease progression. These proteins could potentially make significant contributions to the prediction of aggressive metastatic disease compared to non-metastatic primary tumors. In addition they could assist in developing better treatment strategies for the disease. Further studies are needed to uncover the mechanisms responsible for these proteins in the development and progression of prostate cancer.

## Supporting Information

Figure S1 **Experimental work flow.** Schematic diagram summarizing the iTRAQ labeling, digestion, fractionation and LC-MALDI-MS/MS analysis of PC3-N2 and PC3-ML2 proteins.
(TIF)

Figure S2 **Representative tandem mass spectra for a Plectin (Panel A), Vimentin (Panel B), GAPDH (Panel C) and EphA2 (Panel D) peptides and inserts showing the peak area at the low mass/charge (m/z) region with the iTRAQ reporter ions.** Insert tables shows the masses of the b and y ion series.
(TIF)

Figure S3 **Ingenuity pathway analysis showing top interaction network.** Ingenuity pathway analysis was used to assemble a network based upon the differentially expressed proteins in PC3-ML2 compared to PC3-N2. The protein names are given in Table S1.
(TIF)

Figure S4 **Confocal microscopy of PC3-ML2 cells for visualization of vimentin.** Cells were treated with control siRNA or vimentin gene specific siRNA for 3 days and then fixed, incubated with mouse monoclonal anti-vimentin primary antibodies and "stained" with Alexa Fluor 488 conjugated goat anti-mouse secondary antibodies (green) and the nuclear stain TOPRO (blue).
(TIF)

Figure S5 **Cell viability assay PC3-ML2 cells.** Control siRNA, plectin siRNA and vimentin siRNA respectively were used knockdown PC3-ML2 cells before performing cell viability assays. There are no significant differences between the viability of the control and the plectin and vimentin knockdown cells.
(TIF)

Figure S6 **Expression of plectin and vimentin in PC3 and RWPE-1 cells.** Total cell lysates (40 µg) of PC3 and RWPE-1 cells were subjected to SDS-PAGE. The separated proteins were analyzed by Western blot analysis to detect plectin as described. GAPDH detection was included as a loading control.
(TIF)

Table S1 **Proteins that are differentially regulated (expressed) between PC3-ML2 vs PC3-N2 cells.** These proteins show an averaged ratio-fold change ≥1.5 or≤0.667 in the duplicate experiments between the two cell lines (*t* test, *p*<0.05).
(DOCX)

Table S2 **Ingenuity knowledge base analysis showing the top biological functions of the differentially regulated proteins between PC3-ML2 and PC3-N2 cells, the probability scores and the number of molecules in the functional category.**
(DOCX)

Table S3 **Ingenuity knowledge base analysis showing the top biological functions of the differentially regulated proteins between PC3-ML2 and PC3-N2 cells, the probability scores and the number of molecules in each disease category and disorder.**
(DOCX)

Table S4 **Ingenuity knowledge base analysis showing the top biological functions of the differentially regulated proteins between PC3-ML2 and PC3-N2 cells, the probability scores and the top IPA.**
(DOCX)
