# Supplementary figures and images for: Variable Metastatic Potentials Correlate with Differential Plectin and Vimentin Expression in Syngeneic Androgen Independent Prostate Cancer Cells
Source: PLoS One. 2013 May 22;8(5):e65005. doi: 10.1371/journal.pone.0065005 (PMC3661497; doi:10.1371/journal.pone.0065005)

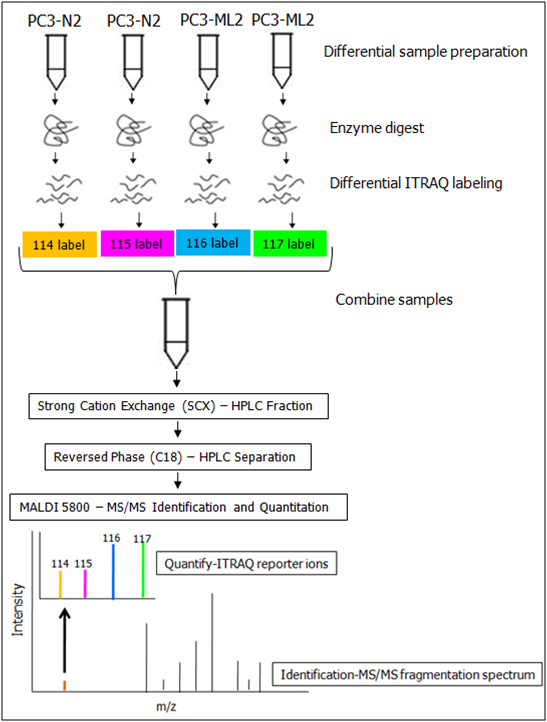

Supplement: Figure S1 — Experimental work flow. Schematic diagram summarizing the iTRAQ labeling, digestion, fractionation and LC-MALDI-MS/MS analysis of PC3-N2 and PC3-ML2 proteins. (TIF) [file pone.0065005.s001.tif]

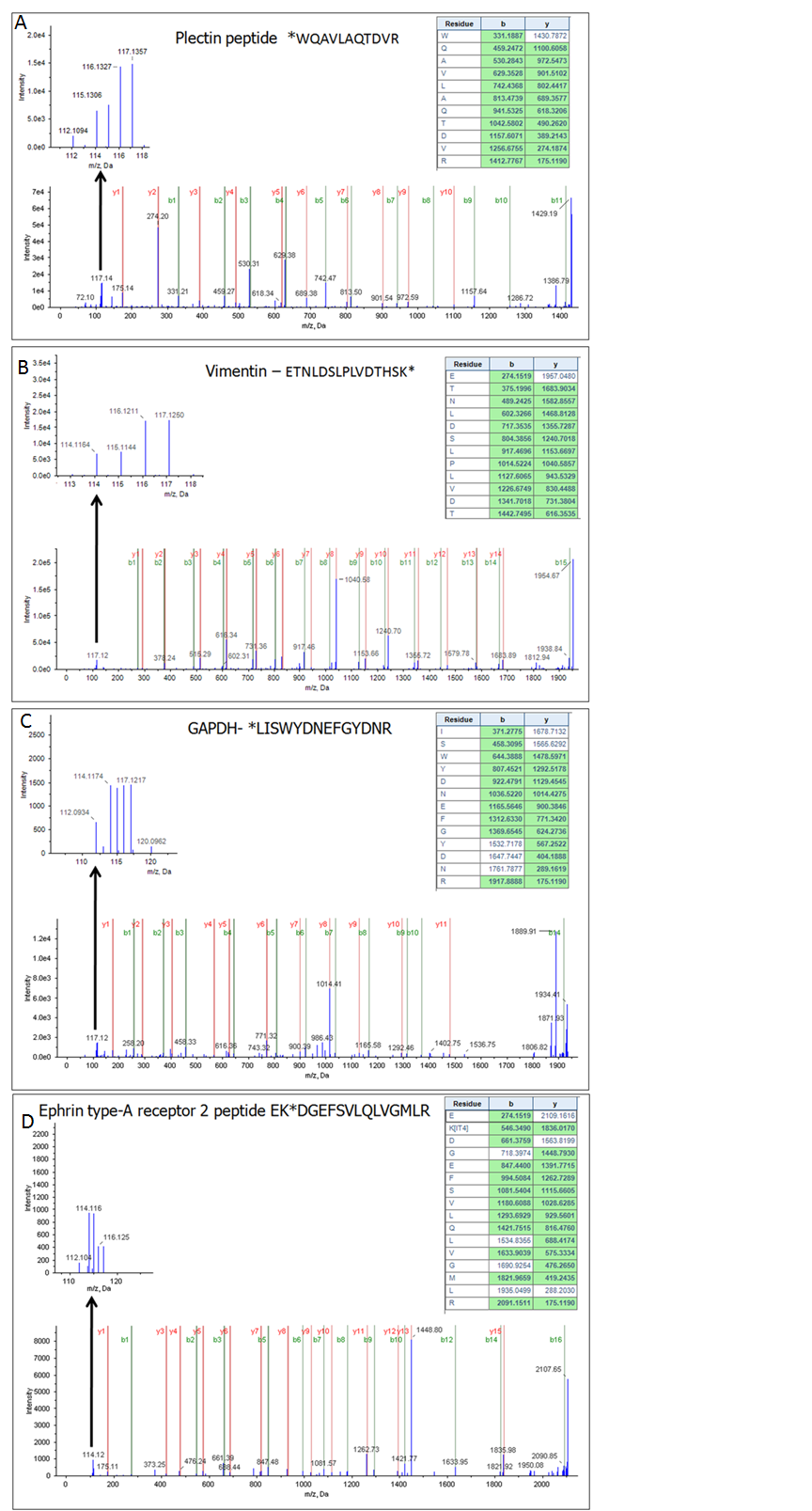

Supplement: Figure S2 — Representative tandem mass spectra for a Plectin (Panel A), Vimentin (Panel B), GAPDH (Panel C) and EphA2 (Panel D) peptides and inserts showing the peak area at the low mass/charge (m/z) region with the iTRAQ reporter ions. Insert tables shows the masses of the b and y ion series. (TIF) [file pone.0065005.s002.tif]

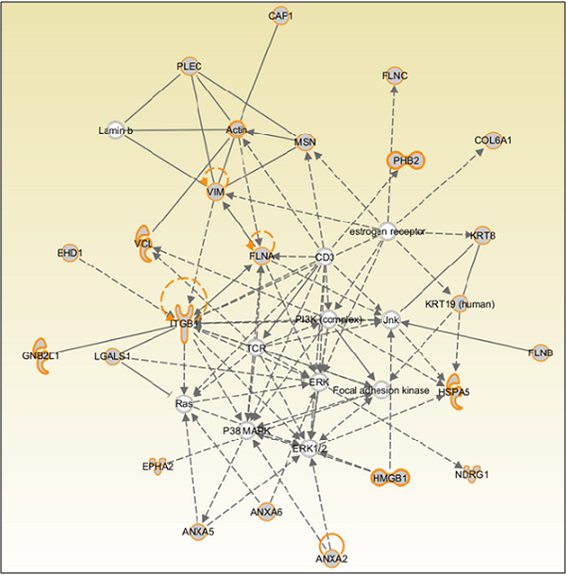

Supplement: Figure S3 — Ingenuity pathway analysis showing top interaction network. Ingenuity pathway analysis was used to assemble a network based upon the differentially expressed proteins in PC3-ML2 compared to PC3-N2. The protein names are given in Table S1. (TIF) [file pone.0065005.s003.tif]

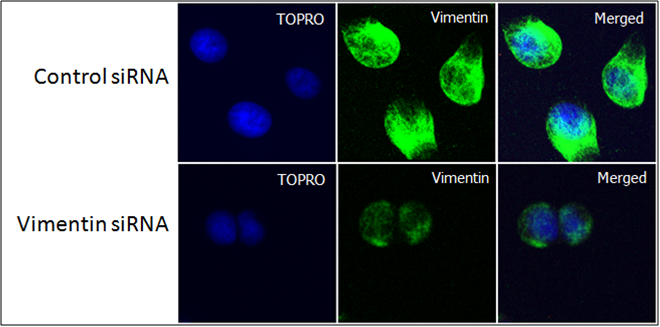

Supplement: Figure S4 — Confocal microscopy of PC3-ML2 cells for visualization of vimentin. Cells were treated with control siRNA or vimentin gene specific siRNA for 3 days and then fixed, incubated with mouse monoclonal anti-vimentin primary antibodies and "stained" with Alexa Fluor 488 conjugated goat anti-mouse secondary antibodies (green) and the nuclear stain TOPRO (blue). (TIF) [file pone.0065005.s004.tif]

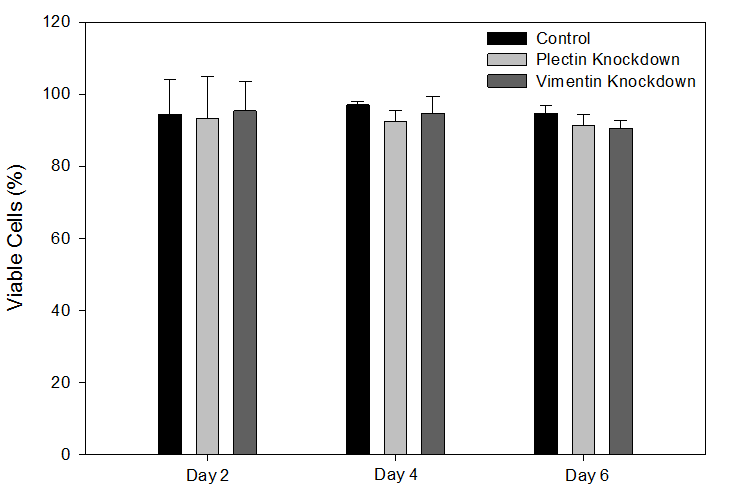

Supplement: Figure S5 — Cell viability assay PC3-ML2 cells. Control siRNA, plectin siRNA and vimentin siRNA respectively were used knockdown PC3-ML2 cells before performing cell viability assays. There are no significant differences between the viability of the control and the plectin and vimentin knockdown cells. (TIF) [file pone.0065005.s005.tif]

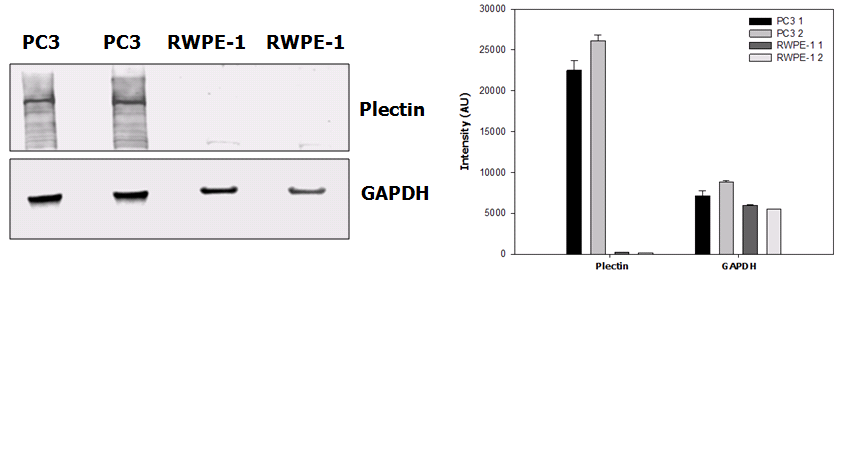

Supplement: Figure S6 — Expression of plectin and vimentin in PC3 and RWPE-1 cells. Total cell lysates (40 µg) of PC3 and RWPE-1 cells were subjected to SDS-PAGE. The separated proteins were analyzed by Western blot analysis to detect plectin as described. GAPDH detection was included as a loading control. (TIF) [file pone.0065005.s006.tif]
