# Supplementary material for: Variable Metastatic Potentials Correlate with Differential Plectin and Vimentin Expression in Syngeneic Androgen Independent Prostate Cancer Cells
Source: PLoS One. 2013 May 22;8(5):e65005. doi: 10.1371/journal.pone.0065005 (PMC3661497; doi:10.1371/journal.pone.0065005)
Supplement: Table S1 — Proteins that are differentially regulated (expressed) between PC3-ML2 vs PC3-N2 cells. These proteins show an averaged ratio-fold change ≥1.5 or≤0.667 in the duplicate experiments between the two cell lines (t test, p<0.05). (DOCX) [file pone.0065005.s007.docx]

|  |  |  | **Table S1. Differentially Regulated Proteins** |  |  |  |
| --- | --- | --- | --- | --- | --- | --- |
|  |  |  |  |  |  |  |
| # | **Accession** | **Gene** | **Protein Description** | **Number of** | **% Sequence** | **Mean ITRAQ Ratio** |
|  | **#** | **Symbol** |  | **Peptides** | **Coverage** | **ML2/N2** |
| 1 | Q15149 | PLEC | Plectin | 170 | 39.8 | 8.47 |
| 2 | P63261 | ACTG1 | Actin, cytoplasmic 2 | 122 | 60 | 0.144 |
| 3 | Q86XU5 | MYH9 | Myosin, heavy chain 9, non-muscle | 110 | 52.2 | 2.1 |
| 4 | P21333 | FLNA | Filamin-A | 81 | 41 | 1.905 |
| 5 | O75369 | FLNB | Filamin-B | 75 | 35.7 | 0.16 |
| 6 | P12814 | ACTN1 | Actinin, alpha 1 | 74 | 56.4 | 2.7 |
| 7 | P49327 | FASN | Fatty acid synthase | 54 | 29 | 0.597 |
| 8 | P68104 | EEF1A1 | Elongation factor 1-alpha 1 | 54 | 69 | 0.413 |
| 9 | Q49AL0 | CLTC | Clathrin heavy chain 1 | 54 | 36 | 0.366 |
| 10 | P08670 | VIM | Vimentin | 52 | 72 | 6.19 |
| 11 | Q6P2H7 | DYNC1H1 | Cytoplasmic dynein 1 heavy chain 1 | 48 | 13 | 0.57 |
| 12 | P00558 | PGK1 | Phosphoglycerate kinase 1 | 46 | 68 | 3.87 |
| 13 | P07355 | ANXA2 | Annexin A2 | 45 | 64.4 | 0.424 |
| 14 | Q14917 | SPTAN1 | Spectrin alpha chain, brain isoform 1 | 44 | 23 | 2.2 |
| 15 | P26038 | MSN | Moesin | 42 | 53 | 0.34 |
| 16 | P10809 | HSPD1P1 | 60 kda heat shock protein, mitochondrial | 42 | 61 | 0.313 |
| 17 | P11021 | HSPA5 | 78 kda glucose-regulated protein | 39 | 55 | 2.33 |
| 18 | P18206 | VCL | Vinculin | 36 | 39 | 1.72 |
| 19 | P02545 | LMNA | Lamin A/C | 35 | 47 | 2.8 |
| 20 | P08107 | HSPA1A | Heat shock 70 kda protein 1A/1B | 35 | 52 | 1.819 |
| 21 | P30101 | PDIA3 | Protein disulfide-isomerase | 34 | 57 | 5.15 |
| 22 | P08133 | ANXA6 | Annexin A6 | 34 | 48 | 1.5 |
| 23 | P22392 | NME2 | Nucleoside diphosphate kinase B | 32 | 83 | 4.52 |
| 24 | P60174 | TPI1 | Triosephosphate isomerase 1 | 31 | 88 | 2.65 |
| 25 | Q14315 | FLNC | Filamin-C | 31 | 18 | 0.15 |
| 26 | Q14974 | KPNB1 | Karyopherin (importin) beta 1 | 30 | 30.1 | 0.529 |
| 27 | P62826 | RAN | GTP-binding nuclear protein Ran | 26 | 47 | 0.505 |
| 28 | P06576 | ATP5B | ATP synthase subunit beta, mitochondrial | 24 | 54 | 2.44 |
| 29 | Q06830 | PRDX1 | Peroxiredoxin-1 | 24 | 81.4 | 2.44 |
| 30 | P30041 | PRDX6 | Peroxiredoxin-6 | 24 | 74.1 | 1.75 |
| 31 | P25705 | ATP5a1 | ATP synthase subunit alpha, mitochondrial | 24 | 54.1 | 1.513 |
| 32 | P60842 | EIF4A1 | Eukaryotic initiation factor 4A-I | 24 | 49 | 0.424 |
| 33 | Q13200 | PSMD2 | 26S proteasome non-ATPase regulatory subunit 2 | 23 | 30 | 0.602 |
| 34 | P08727 | KRT19 | Keratin 19 | 22 | 51 | 0.4 |
| 35 | P08758 | ANXA5 | Annexin A5 | 21 | 69 | 7.44 |
| 36 | Q15942 | ZYX | Zyxin | 21 | 47 | 1.77 |
| 37 | Q04637 | EIF4G1 | Eukaryotic translation initiation factor 4 gamma 1 | 20 | 16 | 0.586 |
| 38 | P09382 | LGALS1 | Galectin-1 | 19 | 65.2 | 2.44 |
| 39 | Q96IH1 | FSCN1 | Fascin | 19 | 35.9 | 2.29 |
| 40 | P02786 | TFRC | Transferrin receptor (p90, CD71) | 18 | 27.6 | 1.999 |
| 41 | P34932 | HSPA4 | Heat shock 70 kda protein 4 | 18 | 26.9 | 0.63 |
| 42 | P20073 | ANXA7 | Annexin A7 | 17 | 29.3 | 1.659 |
| 43 | Q6P1L4 | PYGL | Glycogen phosphorylase | 16 | 30 | 2.77 |
| 44 | P06737 | PYGP | Glycogen phosphorylase, brain isoform | 16 | 30.2 | 1.94 |
| 45 | Q92598 | HSPH1 | Heat shock protein 105 kda | 16 | 24.2 | 0.44 |
| 46 | Q6FHU3 | PSME1 | Proteasome activator subunit 1 | 15 | 42.6 | 5.34 |
| 47 | P29401 | TKT | Transketolase | 15 | 3.04 | 3.04 |
| 48 | P49915 | GMPS | Guanine monophosphate synthetase | 15 | 25.2 | 0.597 |
| 49 | P63244 | GNB2L1 | Guanine nucleotide binding protein (G protein) | 15 | 62.2 | 0.519 |
| 50 | Q02790 | FKBP4 | Peptidyly-prolyl-cis trans isomerase | 15 | 37.9 | 0.483 |
| 51 | Q969I0 | KRT8 | Keratin, type II cytoskeletal 8 | 15 | 36.8 | 0.478 |
| 52 | Q96C96 | P4HB | Prolyl 4-hydroxylase, beta polypeptide | 14 | 28.7 | 1.73 |
| 53 | P55209 | NAP1L1 | Nucleosome assembly protein 1-like 1 | 14 | 31 | 1.659 |
| 54 | Q9H4M9 | EHD1 | EH-domain containing 1 | 14 | 52.1 | 0.63 |
| 55 | Q01518 | CAP1 | Adenylyl cyclase-associated protein 1 | 14 | 37.3 | 0.42 |
| 56 | Q12931 | TRAP1 | Heat shock protein 75 kda, mitochondrial | 13 | 26.3 | 0.457 |
| 57 | P45880 | VDAC2 | Voltage-dependent anion-selective channel protein 2 | 13 | 49.7 | 0.316 |
| 58 | P37802 | TAGLN2 | Transgelin-2 | 12 | 65 | 1.83 |
| 59 | Q02952 | AKAP12 | A kinase (PRKA) anchor protein 12 | 12 | 9.1 | 0.487 |
| 60 | A7E2S3 | RPS3 | Ribosomal protein S3 | 12 | 39.4 | 0.474 |
| 61 | Q9BVS9 | IPO5 | Importin 5 | 12 | 14.9 | 0.44 |
| 62 | P78527 | PRKDC | DNA-dependent protein kinase catalytic subunit | 12 | 3.5 | 0.44 |
| 63 | Q08J23 | NSUN2 | tRNA cytosine methyltransferase | 11 | 20.2 | 1.599 |
| 64 | Q6NUS1 | PDCD6IP | Programmed cell death 6-interacting protein | 11 | 14.4 | 0.524 |
| 65 | Q99832 | CCT7 | Chaperonin containing TCP1, subunit 7 | 11 | 24.9 | 0.469 |
| 66 | P62906 | RPL10AP9 | Ribosomal protein l10a | 11 | 47.2 | 0.405 |
| 67 | P80723 | BASP1 | Brain acid soluble protein 1 | 11 | 78 | 0.26 |
| 68 | P62158 | CALM | Calmodulin | 10 | 58.4 | 2 |
| 69 | Q05639 | EEF1A2 | Eukaryotic translation elongation factor 1 alpha 2 | 10 | 53.8 | 3.597 |
| 70 | P53999 | SUB1 | SUB1 homolog | 9 | 47.2 | 2.679 |
| 71 | P00491 | PNP | Purine nucleoside phosphorylase | 9 | 36 | 2.39 |
| 72 | Q16543 | CDC37 | Hsp90 co-chaperone Cdc37 | 9 | 27.3 | 2.33 |
| 73 | P23381 | WARS | Tryptophanyl-tRNA synthetase | 9 | 24.6 | 1.87 |
| 74 | Q3KQZ8 | EPRS | Glutamyl-prolyl-tRNA synthetase | 9 | 17.3 | 0.654 |
| 75 | Q6P2Q9 | PRPF8 | Pre-mRNA-processing-splicing factor 8 | 9 | 5.5 | 0.501 |
| 76 | Q15067 | ACOX1 | Acyl-Coenzyme A oxidase 1 | 8 | 20.2 | 6.606 |
| 77 | Q05BT9 | COL6A1 | Collagen, type VI, alpha 1 | 8 | 9.4 | 3.3 |
| 78 | Q9NRW3 | APOBEC3C | Apolipoprotein B mRNA editing enzyme | 8 | 36 | 2.2 |
| 79 | Q00688 | FKBP3 | Peptidyl-prolyl cis-trans isomerase FKBP3 | 8 | 31.7 | 2.1 |
| 80 | P62917 | RPL8 | Ribosomal protein L8 | 8 | 30 | 0.469 |
| 81 | O43143 | DHX15 | Pre-mRNA-splicing factor ATP-dep. RNA helicase | 8 | 15.9 | 0.36 |
| 82 | Q9NTK5 | OLA1 | Obg-like ATPase 1 | 8 | 25.8 | 0.353 |
| 83 | Q8N165 | PDLIM1 | PDZ and LIM domain protein 1 | 7 | 35.6 | 2.39 |
| 84 | Q9BSJ8 | ESYT1 | Extended synaptotagmin-1 | 7 | 8.8 | 2.376 |
| 85 | Q9UL46 | PSME2 | Proteasome activator complex subunit 2 | 7 | 27.2 | 2.26 |
| 86 | P40925 | MDH1 | Malate dehydrogenase 1 | 7 | 21.6 | 2.26 |
| 87 | O14980 | XPO1 | Exportin-1 | 7 | 9.7 | 0.544 |
| 88 | Q05CK9 | SYNCRIP | Synaptotagmin binding, cytoplasmic RNA interacting protein | 7 | 8.8 | 0.505 |
| 89 | Q15366 | PCBP2 | Poly(rc) binding protein 2 | 7 | 32.4 | 0.487 |
| 90 | P21796 | VDAC1P1 | Voltage-dependent anion-selective channel protein 1 | 7 | 23.3 | 0.478 |
| 91 | P53618 | COPB1 | Coatomer subunit beta | 7 | 10.3 | 0.366 |
| 92 | Q99623 | PHB2 | Prohibitin-2 | 7 | 24.8 | 0.26 |
| 93 | P13796 | LCP1 | Plastin-2 | 7 | 25.8 | 0.03 |
| 94 | O60701 | UGDH | UDP-glucose 6-dehydrogenase | 6 | 18.7 | 5.05 |
| 95 | P05556 | ITGB1 | Integrin, beta 1 | 6 | 11 | 4.786 |
| 96 | O00299 | CLIC1 | Chloride intracellular channel 1 | 6 | 34.9 | 2.37 |
| 97 | Q6IBN6 | CBX1 | Chromobox protein homolog 1 | 6 | 35.6 | 2.22 |
| 98 | P61353 | RPL27 | Ribosomal protein L27 | 6 | 46.3 | 0.496 |
| 99 | P12429 | ANXA3 | Annexin A3 | 6 | 23.5 | 0.34 |
| 100 | P52597 | HNRNPF | Heterogeneous nuclear ribonucleoprotein F | 6 | 26 | 0.322 |
| 101 | Q96KP4 | CNDP2 | Cytosolic non-specific dipeptidase 2 | 6 | 17.7 | 0.159 |
| 102 | O95573 | ACSL3 | Long-chain-fatty-acid-CoA ligase 3 | 6 | 9.3 | 0.131 |
| 103 | Q04760 | GLO1 | Glyoxalase I | 5 | 38.6 | 6.02 |
| 104 | P40121 | CapG | Macrophage-capping protein | 5 | 19 | 4.74 |
| 105 | P54819 | AK2 | Adenylate kinase 2 | 5 | 32.5 | 1.836 |
| 106 | P09429 | HMGB1 | High mobility group protein B1 | 5 | 39.6 | 0.597 |
| 107 | Q9HB71 | CACYBP | Calcyclin-binding protein | 5 | 39.5 | 0.4 |
| 108 | Q27J81 | INF2 | Inverted formin-2 | 5 | 6.4 | 0.272 |
| 109 | P04183 | TK1 | Thymidine kinase, soluble | 4 | 22 | 8.7 |
| 110 | Q92597 | NDRG1 | Protein NDRG1 | 4 | 18 | 6.36 |
| 111 | P07196 | NEFL | Neurofilament light polypeptide | 4 | 9 | 5.86 |
| 112 | Q5BJH1 | PSAP | Prosaposin | 4 | 5.39 | 5.39 |
| 113 | P46459 | NSF | N-ethylmaleimide-sensitive factor | 4 | 5.8 | 4.36 |
| 114 | O95678 | KRT75 | Keratin, type II cytoskeletal 75 | 4 | 12.3 | 3.597 |
| 115 | Q9Y224 | CLE | Chromosome 14 open reading frame 166 (UPF0568 protein) | 4 | 21.3 | 3.13 |
| 116 | Q14152 | EIFA3 | Eukaryotic translation initiation factor 3 subunit A | 4 | 12 | 0.505 |
| 117 | P39748 | FEN1 | Flap endonuclease 1 | 4 | 4 | 0.349 |
| 118 | P29317 | EPHA2 | Ephrin type-A receptor 2 | 4 | 7.8 | 0.299 |
| 119 | Q15645 | TRIP13 | Thyroid hormone receptor interactor 13 | 4 | 5.9 | 0.011 |
| 120 | Q92890 | UFD1l | Ubiquitin fusion degradation 1 like | 3 | 11.7 | 1.8 |
| 121 | Q9Y2A9 | TMEM3 | Transmembrane protein 43 | 3 | 14.7 | 4.57 |
| 122 | O15118 | NPC1 | Niemann-Pick C1 protein | 3 | 2.72 | 2.72 |
| 123 | P63173 | RPL38 | Ribosomal protein L38 | 3 | 35.7 | 0.613 |
| 124 | Q8TCJ2 | STT3b | STT3, subunit of the oligosaccharyltransferase complex | 3 | 6.2 | 0.328 |
| 125 | P22059 | OSBP1 | Oxysterol-binding protein | 2 | 3 | 8.31 |
| 126 | Q2TAY7 | SMU1 | Smu-1 suppressor of mec-8 and unc-52 homolog | 2 | 6.6 | 6.7 |
| 127 | P33992 | MCM5 | Minichromosome maintenance complex comp. 5 | 2 | 4 | 5.05 |
| 128 | Q96C90 | PNG | Protein phosphatase 1 regulatory subunit 14B | 2 | 17 | 2.7 |
| 129 | P61006 | RAB8A | Ras-related protein Rab-8A | 2 | 12.6 | 0.013 |
| 130 | Q9UPN7 | PPP6R1 | Serine/threonine-protein phosphatase 6 regulatory subunit 1 | 2 | 2.8 | 0.0124 |
