# Supplementary material for: Variable Metastatic Potentials Correlate with Differential Plectin and Vimentin Expression in Syngeneic Androgen Independent Prostate Cancer Cells
Source: PLoS One. 2013 May 22;8(5):e65005. doi: 10.1371/journal.pone.0065005 (PMC3661497; doi:10.1371/journal.pone.0065005)
Supplement: Table S2 — Ingenuity knowledge base analysis showing the top biological functions of the differentially regulated proteins between PC3-ML2 and PC3-N2 cells, the probability scores and the number of molecules in the functional category. (DOCX) [file pone.0065005.s008.docx]

|  | **Table S2. Top Biological functions** | |  |
| --- | --- | --- | --- |
|  |  |  |  |
| **#** | **Molecular and Cellular Functions** | **p- value** | **# Molecules** |
| 1 | Cellular Growth and Proliferation | 9.13E-10 - 4.76E-02 | 38 |
| 2 | Cell Morphology | 1.01E-05 - 4.62E-02 | 21 |
| 3 | Nucleic Acid Metabolism | 5.85E-05 - 3.98E-02 | 14 |
| 4 | Cellular Assembly and Organization | 6.48E-05 - 4.32E-02 | 27 |
| 5 | Cell Death | 1.53E-05 - 4.76E-02 | 34 |
