# Supplementary material for: Variable Metastatic Potentials Correlate with Differential Plectin and Vimentin Expression in Syngeneic Androgen Independent Prostate Cancer Cells
Source: PLoS One. 2013 May 22;8(5):e65005. doi: 10.1371/journal.pone.0065005 (PMC3661497; doi:10.1371/journal.pone.0065005)
Supplement: Table S3 — Ingenuity knowledge base analysis showing the top biological functions of the differentially regulated proteins between PC3-ML2 and PC3-N2 cells, the probability scores and the number of molecules in each disease category and disorder. (DOCX) [file pone.0065005.s009.docx]

|  | **Table S3. Top Diseases and Disorders** | | |  | |
| --- | --- | --- | --- | --- | --- |
|  |  |  | |  | |
| **#** | **Diseases and Disorders** | **p- value** | | **# Molecules** | |
| 1 | Cancer | 1.11E-07 - 4.94E-02 | 68 | |  |
| 2 | Dermatological Diseases and Conditions | 1.52E-06 - 4.62E-02 | | 35 | |
| 3 | Genetic Disorder | 1.52E-06 - 3.98E-02 | | 60 | |
| 4 | Gastrointestinal Disease | 5.96E-06 - 2.14E-02 | | 32 | |
| 5 | Immunological Disease | 1.98E-05 - 4.76E-02 | | 17 | |
