# Supplementary material for: Variable Metastatic Potentials Correlate with Differential Plectin and Vimentin Expression in Syngeneic Androgen Independent Prostate Cancer Cells
Source: PLoS One. 2013 May 22;8(5):e65005. doi: 10.1371/journal.pone.0065005 (PMC3661497; doi:10.1371/journal.pone.0065005)
Supplement: Table S4 — Ingenuity knowledge base analysis showing the top biological functions of the differentially regulated proteins between PC3-ML2 and PC3-N2 cells, the probability scores and the top IPA. (DOCX) [file pone.0065005.s010.docx]

|  | **Table S4. Top IPA Networks** |  |
| --- | --- | --- |
|  |  |  |
| **#** | **Top IPA Networks** | **Score** |
| 1 | Cell Morphology, Connective Tissue Development and Function, Cellular Movement. | 37 |
| 2 | Amino Acid Metabolism, Post-Translational Modification, Small Molecule Biochemistry. | 20 |
| 3 | Cellular Assembly & Organization, DNA Replication, Recombination, Repair, Cellular Compromise. | 18 |
| 4 | Cell Morphology, Cellular Compromise, DNA Replication, Recombination, and Repair. | 16 |
| 5 | Antigen Presentation, Cellular Movement, Hematological System Development and Function. | 16 |
